# Self-Organization of the Biological Evolution

**DOI:** 10.3390/genes10110854

**Published:** 2019-10-28

**Authors:** Werner Arber

**Affiliations:** Biozentrum, University of Basel, Klingelbergstrasse 70, CH-4056 Basel, Switzerland; werner.arber@unibas.ch

**Keywords:** genetic variants, natural selection, *E. coli* bacteria, restriction and modification, restriction endonucleases, in vivo and in vitro horizontal gene transfer, genetically modified organisms (GMOs), point mutants, mobile genetic elements, evolution genes, duality of the genome, permanent creation, tree of evolution

## Abstract

We report here experiments carried out with nonpathogenic *Escherichia coli* bacterial strains and their phages. This research yielded interesting insights into their activities, occasionally producing genetic variants of different types. In order to not interfere with the genetic stability of the parental strains involved, we found that the bacteria are genetically equipped to only rarely produce a genetic variant, which may occur by a number of different approaches. On the one hand, the genes of relevance for the production of specific genetic variants are relatively rarely expressed. On the other hand, other gene products act as moderators of the frequencies that produce genetic variants. We call the genes producing genetic variants and those moderating the frequencies of genetic variation “evolution genes”. Their products are generally not required for daily bacterial life. We can, therefore, conclude that the bacterial genome has a duality. Some of the bacterial enzymes involved in biological evolution have become useful tools (e.g., restriction endonucleases) for molecular genetic research involving the genetic set-up of any living organism.

Classical investigations of biological evolution were usually based on the phenotypes of eukaryotic organisms. This allowed Charles Darwin to propose the theory of natural selection, stating that organisms can occasionally produce phenotypic variants able to live under altered living conditions. More recently, it became obvious that life activities are rendered possible by the expression of genetic information carried on filamentous DNA molecules. Experimental investigations with microorganisms opened new approaches to a more detailed understanding, both of the production of genetic variants and of the occurrence of natural selection.

In this piece, I review research that I conducted, together with my collaborators, in the second half of the 20th century. Our studies were mainly carried out with nonpathogenic bacterial strains of *E. coli* and with their viruses, called bacteriophages, or, for short, phages. These bacteria generally carry their genetic information on a single circular DNA filament. If we compare one nucleotide pair with one letter in a book, the size of the genome of *E. coli* roughly corresponds to the size of the Bible. We can conclude that, in general, the bacterial genomes are about the size of a book. In addition, bacteria can sometimes carry plasmids, which are circular DNA molecules roughly equivalent to one page.

Genetic variation by horizontal gene transfer has been known to occasionally occur between different mutants of a bacterial strain and by a few processes:

(1) In transformation, a free segment of bacterial DNA can become absorbed by a living bacterial cell.
(2) In conjugation, DNA from a bacterial cell can become transferred to another variant cell by cell–cell contact with the help of a fertility plasmid.
(3) In transduction an infectious phage particle carrying a fraction of a bacterial genome can inject its DNA, after adsorption, into another kind of bacterial cell.

Some phages can lysogenize a host cell either by integrating the phage genome into the host genome (e.g., phage lambda) or by forming a plasmid with its genome, which then becomes replicated in synchrony with the bacterial propagation (e.g., phage P1). When phage reproduction by these lysogenic bacteria becomes induced, some of the progeny phage particles can contain a small section of the host genome. When an appropriate bacterial mutant strain becomes infected by this phage population, some of the surviving host cells can carry in their genome the genetic bacterial information from the transducing phages. Transduction is, of course, only possible if the bacteria concerned allow specific adsorption of the transducing phage particle.

In using two related host strains for phage propagation, the phenomenon “host-controlled restriction and modification” can be described as follows: A phage produced on its usual host strain can also infect a related host bacteria, but this alternative host may only reproduce very few progeny phage particles (called restriction). These progeny phages can then efficiently become reproduced in their last host, but no longer in their original host bacteria. The reproduced phage particles become modified in their originally restricting host. By labelling the phage DNA with radioactive P^32 ^we showed in 1960 that restriction can be explained by rendering the infecting DNA acid-soluble. We also showed that any foreign DNA became degraded upon penetration into a restricting host, such as by transformation, conjugation and transduction.

Restriction endonucleases were isolated in the 1970s. Protection from degradation of the host DNA and of modified phage DNA was brought about by site-specific methylation, an epigenetic phenomenon. In the meantime, it has become known that specific restriction and modification systems are present on the genomes of thousands of, often, related bacterial strains, and also on some plasmids and phage genomes, such as phage P1. Not all identified restriction enzymes cut infecting foreign DNA at their short specific nucleotide sequences, identified by the restricting host for cutting the foreign DNA and for protecting the cells own genome by methylation at their specificity sites.

In the meantime, restriction enzymes of type II cutting foreign DNA molecules reproducibly at their short unmethylated recognition sites have become a very convenient tool for structural and functional studies on isolated DNA segments. Therefore, acquired knowledge can, among other things, serve for the transfer of a well-known DNA segment into the genome of another organism. This kind of experimental horizontal gene transfer can efficiently render a selected kind of organism useful for practical applications after a careful study of the properties of the genetically modified organism (GMO).

In this context it is interesting that we can also conclude that restriction-modification systems serve, in nature, to considerably reduce the efficiency of naturally occurring horizontal gene transfer. One should remember that biological evolution depends on the occasional production of genetic variants and on their natural usefulness upon natural selection. Several natural processes have become known to occasionally produce a genetic variant. While processes of horizontal gene transfer are steps to render a genetic function available for a functional improvement of other living organisms, other natural processes can mutagenize already existing DNA segments on their genome. This process can occasionally also improve their functional capacities. One of these processes can produce point mutations. Nucleotide structures are not entirely stable and can occasionally produce short-living isomeric forms. Such nucleotide derivatives can integrate an alternative nucleotide upon DNA replication. Some such nascent mutations can escape repair processes and yield a nucleotide substitution in the genome. Other mutagenesis processes can involve the translocation of a so-called mobile genetic element to another site in the genome. This can result in both, a deletion and an insertion at various sites of the genome. Mobile genetic elements can sometimes also produce duplications or other genetic variants. Specific enzymes are generally involved in these processes and these enzymes are rarely available. Nature takes care to maintain the genomic structure, although occasionally producing a genetic variant.

Of the available results from experimental investigations, we can make the following assumptions: Bacterial cells are genetically equipped to relatively rarely produce a genetic variant at various sites of the genome. For these processes they can occasionally express specific genes. Their products serve the mutagenesis process in question. These genes serve to exclusively promote biological evolution. We therefore call them evolution genes. Other evolution genes produce products that seriously modulate the frequency of genetic variation. An example of this modulation is the function of restriction and modification systems.

The evolution genes described here are located on the genome of the bacterial cell. We can conclude that this genome has a duality. Most genes serve the expression of life functions of the cell, whereas only a few genes (evolution genes) ensure the biological evolution of the species in question by paying attention to the genetic variation that occurs only rarely. We can conclude that nature takes active care to occasionally produce novel genetic variants that are then tested by natural selection on their capacity to propagate under alternative living conditions. Our present scientific insights point to the wonderful system of permanent creation by the biological evolution carried out by the activities of evolution genes. We can conclude that biological evolution is a system of self-organization.

Archeological investigations suggest that our sun and its planets originated about 4000 million years ago, and that unicellular microorganisms were already present on planet Earth about 3500 million years ago. We can assume that this may also have been the start of slowly progressing biological evolution by permanent creation. So far, it has not been possible to explain the creation of the first living microbial cell.

The classical tree of biological evolution drawn by Charles Darwin suggests that all living organisms presently in existence on our planet have a common origin. In view of the contribution of horizontal gene transfer to the ongoing biological evolution, we can symbolically draw horizontal connections between different branches of the tree of evolution. Since horizontal gene transfer is likely to continue to contribute in the future to biological evolution, we can conclude that living organisms have not only a common origin but also a common future. For this reason, we should try to avoid a massive loss of the rich biodiversity, which our planet presently possesses.

In the environment, horizontal gene transfer can be assumed to occur between closely cohabiting organisms, for example those in microbiomes. On the other hand, the in vitro preparation of genetically modified organisms (GMOs) has become possible thanks to available biotechnology. Although hybrids can occasionally also spontaneously occur in vivo, it is essential to experimentally investigate the biosafety of GMOs before their use and introduction into the environment. If responsibly accomplished, in vitro gene transfer can sometimes be safer than classical plant breeding using irradiation of the breeding partners.

In view of our improved knowledge on biological evolution, carefully applied biotechnology could enable humanity to benefit from improved food plants, such as golden rice, and from various other carefully tested GMOs, in order to facilitate life and contribute to a safeguarded, rich biodiversity on planet Earth.

